# Phosphatidylcholine Metabolic Transformation and Progression Signature as a Pharmacodynamic Biomarker

**DOI:** 10.18632/oncotarget.121

**Published:** 2010-07-14

**Authors:** Eric O. Aboagye

**Affiliations:** Comprehensive Cancer Imaging Centre, Department of Surgery and Cancer, Faculty of Medicine, Imperial College London, Hammersmith Hospital Campus, London W12 0NN, UK

**Keywords:** Hsp90, PTPS, magnetic resonance spectroscopy, melanoma, pharmacodynamic biomarker

The value of biomarkers that report on the effects of drugs at the target site – largely non-invasive/imaging biomarkers - is increasingly being recognised. Such biomarkers can inform whether the drug modulates the target in the desired manner, as well as the magnitude and duration of target modulation and recovery dynamics. Correlative investigations with dynamic contrast-enhanced magnetic resonance imaging (DCE-MRI) and [^18^F]fluorodeoxyglucose positron emission tomography (PET) are routinely employed during first-in-human studies of antiangiogenic/antivascular therapies. There is a broad range of non-invasive imaging/spectroscopic methodologies capable of reporting drug pharmacodynamics at the tumour site [1]. Whereas some imaging/spectroscopic approaches are specific to the biological target protein, others report changes in signalling pathway activity or indeed phenotype.

The ‘phosphatidylcholine transformation and progression signature’ (PTPS) is one such phenotype that can be exploited to assess target drug effect. This signature, which is fairly generic in tumours, has been described extensively for breast cancer [2-7], and to a lesser extent brain [8], prostate [9], ovarian [10] and other cancers. In these cancers, PTPS is characterized by high phosphocholine (PC) levels relative to glycerophosphocholine (GPC), resulting primarily from increased choline kinase activity [4]. PC levels increase in the order senescent mortal cells < cells exhibiting immortalized anchorage-dependent growth < cells exhibiting immortalized anchorage-independent growth < tumorigenic low metastaticity cells < tumorigenic high metastaticity cells; this is analogous to the transformation and progression of normal cells to benign hyperplasia followed by atypical hyperplasia, which progresses to carcinoma *in situ* and finally to distant sites [11]. Growth factor receptor→*Ras*→Erk signal transduction can increase PC levels in cells [2, 12, 13]. Two non-invasive approaches can be used to assess PTPS, namely magnetic resonance spectroscopy (MRS) and PET. PET imaging approaches measure the biosynthesis of PC [11, 12, 14], while MRS-based approaches measure PC derived from both biosynthesis and degradation of phosphatidylcholine; it could be argued, however, that majority of PC results from biosynthesis during cell growth. Hence both nuclear and MRS approaches have the ability to detect the effects of drugs that alter tumor PTPS through inhibition of cell signalling mechanisms or proliferation, the endpoint being a reduction in PC (Figure). Other characteristics of the PTPS are related to increases in GPC levels, and indeed, an early event of transformation is a switch from high GPC/PC ratio to low GPC/PC. High GPC, which may result from the actions of phospholipase A1 and A2 and lysophospholipase, could be considered a reduced malignant phenotype. This latter signature may also result from increased cell death or induction of differentiation, the endpoint being increases in GPC and related free fatty acids (Figure).

**Fig:**
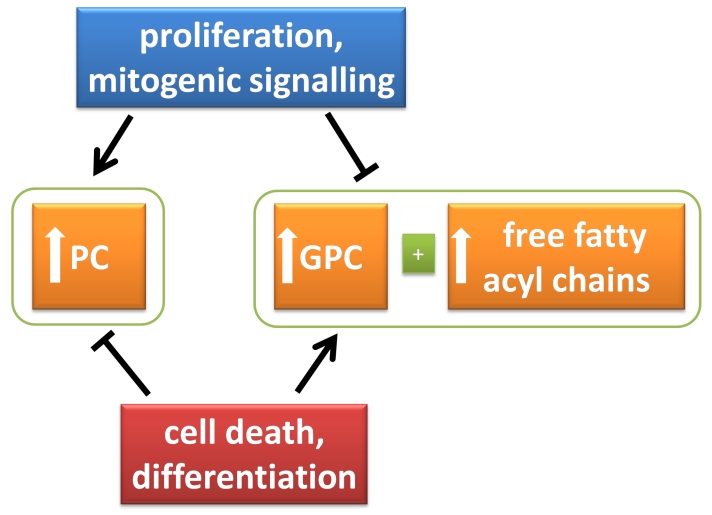
Normalization of the ‘phosphatidylcholine transformation and progression signature’ (PTPS) as a pharmacodynamic biomarker of target tissue drug inhibition. As normal cells transform and progress towards a more malignant phenotype, levels of phosphatidylcholine biosynthetic product, phosphocholine (PC) increases. Proliferation and mitogenic signaling events also increase PC. In contrast, cell death and differentiation, promote degradation of phosphatidylcholine leading increases in the metabolites, glycerophosphocholine (GPC) and associated free fatty acids (detectable as free fatty acyl chains). This phenotype is opposite to an early event in transformation that involves conversion of high GPC/PC ratio to low GPC/PC ratio. Hence drugs that inhibit proliferation, inhibit cell signaling, induce differentiation, or induce cell death could be hypothesized to alter PTPS. In this edition of the Journal, the role of PTPS normalization as a biomarker of Hsp90 inhibition has been investigated by Beloueche-Babari and co-workers.

The work presented by Beloueche-Babari *et al* in this issue accords with the prevailing view on exploitation of PTPS for assessing drug effects. They examined the metabolic response of melanoma cells to inhibition of cell growth by a heat shock protein 90 (Hsp90) inhibitor. Using MRS, they provide proof that, concomitant with growth inhibition and induction of differentiation, the hsp90 inhibitor, 17-AAG, alters the PTPS of melanoma cells in a predictable manner, leading to no changes in PC and increases in GPC, free fatty acyl chains and cytoplasmic lipid droplets. These biochemical changes are consistent with normalization of PTPS. It is often unclear if cellular effects are chemotype-specific (related to the specific structure of drug) or target-specific. In this context they examined a structurally dissimilar Hsp90 inhibitor and showed that the effect was independent of specific Hsp90 inhibitor.

Imaging and spectroscopic methods that exploit the PTPS may have clinical value as target-tissue pharmacodynamic biomarkers in melanoma and other solid tumors. Activating mutations in BRAF occur in ~7% of all human cancers including melanoma, papillary thyroid and colon cancer; the V600E mutation is the most common mutation occurring in approximately 70% of melanoma patients [15]. Activated mutant BRAF binds to an Hsp90-cdc37 complex, which is required for its stability and function. Hsp90 inhibitors disrupt protein stability and, hence, cognate biochemical processes such as MAP kinase signaling. Due to the ability of Hsp90 inhibitors to target several oncogenic proteins, melanomas with activating BRAF mutations, as well as those with WT BRAF activated by NRAS alike are sensitive to inhibitors like 17AAG [16, 17]. Hence, this is a patient population in which drug pharmacology can be objectively assessed and non-responders can be readily identified for alternative therapy. The work by Beloueche-Babari *et al* offers several insights into the potential role of exploiting tumor PTPS normalization as a non-invasive biomarker of anticancer drug effect in general, and specifically, potential use of these methods in melanoma patients for assessing response to Hsp90 inhibitors, as well as other agents like MEK inhibitors that target associated pathways [18, 19]. The study also raises a number of questions around the *in vivo* application of PTPS normalization as a non-invasive biomarker that require further investigation. Standard 31P-MRS methods do not sufficiently resolve individual choline metabolite resonances. Instead composite peaks: phosphomonoesters (comprising PC and phosphoethanolamine) and phosphodiesters (comprising GPC and glycerophosphoethanolamine), are detected in humans MRS measurements [20]. Further studies are, therefore, warranted to determine whether the composite phosphomonoester and phosphodiester resonances are altered with Hsp90 inhibition in melanoma, since these are more clinically assessable measures; the methods have been standardized and reproducibility has been determined in the multi-institutional setting [20]. Alternatively, 1H-decoupled 31P-MRS has shown promise for resolving the metabolite resonances depending on target tissue homogeneity and could be explored for measurement of PTPS normalization [20]. 1H-MRS can be used to assess the tissue levels of CH_2_-free fatty acyl chains that give rise to the MR-visible mobile lipids [21, 22]. Beloueche-Babari *et al* provide a body of evidence, including inhibition of calcium-independent phospholipase A2, to support assessment of these lipid resonances as biomarkers of drug effect. It will be interesting to demonstrate how the free fatty acyl chain resonances change with Hsp90 inhibition *in vivo* in animal models of melanoma and in patients. Beloueche-Babari *et al* demonstrate that the time-dependent evolution of the drug-related changes on growth mirror changes in the biomarker signature. This is important in assigning pharmacological relevance to the biomarker signature. Investigation of the dose-dependence of these PTPS readouts will further support the pharmacological relevance of the biomarker. In this context it will be useful to know whether the spectroscopic readout evolves as all-or-nothing phenomena or are indeed dose-related; the latter will be amenable to investigation of drug effects in early clinical trials of future inhibitors.

In summary, normalization of tumor PTPS appears to be a realistic biomarker for non-invasive monitoring of the pharmacological action of Hsp90 inhibitors. The potential value of this biomarker should be tested *in vivo*.

## References

[1] Workman P, Aboagye EO, Chung YL, Griffiths JR, Hart R, Leach MO, Maxwell RJ, McSheehy PM, Price PM, Zweit J (2006). Minimally invasive pharmacokinetic and pharmacodynamic technologies in hypothesis-testing clinical trials of innovative therapies. J Natl Cancer Inst.

[2] Aboagye EO, Bhujwalla ZM (1999). Malignant transformation alters membrane choline phospholipid metabolism of human mammary epithelial cells. Cancer Res.

[3] Bhujwalla ZM, Aboagye EO, Gillies RJ, Chacko VP, Mendola CE, Backer JM (1999). Nm23-transfected MDA-MB-435 human breast carcinoma cells form tumors with altered phospholipid metabolism and pH: a 31P nuclear magnetic resonance study in vivo and in vitro. Magn Reson Med.

[4] Glunde K, Jie C, Bhujwalla ZM (2004). Molecular causes of the aberrant choline phospholipid metabolism in breast cancer. Cancer Res.

[5] Glunde K, Raman V, Mori N, Bhujwalla ZM (2005). RNA interference-mediated choline kinase suppression in breast cancer cells induces differentiation and reduces proliferation. Cancer Res.

[6] Natarajan K, Mori N, Artemov D, Aboagye EO, Chacko VP, Bhujwalla ZM (2000). Phospholipid profiles of invasive human breast cancer cells are altered towards a less invasive phospholipid profile by the anti-inflammatory agent indomethacin. Adv Enzyme Regul.

[7] Nimmagadda S, Glunde K, Pomper MG, Bhujwalla ZM (2009). Pharmacodynamic markers for choline kinase down-regulation in breast cancer cells. Neoplasia.

[8] Bhakoo KK, Williams SR, Florian CL, Land H, Noble MD (1996). Immortalization and transformation are associated with specific alterations in choline metabolism. Cancer Res.

[9] Ackerstaff E, Pflug BR, Nelson JB, Bhujwalla ZM (2001). Detection of increased choline compounds with proton nuclear magnetic resonance spectroscopy subsequent to malignant transformation of human prostatic epithelial cells. Cancer Res.

[10] Iorio E, Ricci A, Bagnoli M, Pisanu ME, Castellano G, Di Vito M, Venturini E, Glunde K, Bhujwalla ZM, Mezzanzanica D, Canevari S, Podo F Activation of phosphatidylcholine cycle enzymes in human epithelial ovarian cancer cells. Cancer Res.

[11] Contractor KB, Kenny LM, Stebbing J, Al-Nahhas A, Palmieri C, Sinnett D, Lewis JS, Hogben K, Osman S, Shousha S, Lowdell C, Coombes RC, Aboagye EO (2009). [11C]choline positron emission tomography in estrogen receptor-positive breast cancer. Clin Cancer Res.

[12] Liu D, Hutchinson OC, Osman S, Price P, Workman P, Aboagye EO (2002). Use of radiolabelled choline as a pharmacodynamic marker for the signal transduction inhibitor geldanamycin. Br J Cancer.

[13] Ratnam S, Kent C (1995). Early increase in choline kinase activity upon induction of the H-ras oncogene in mouse fibroblast cell lines. Arch. Biochem. Biophys..

[14] Leyton J, Smith G, Zhao Y, Perumal M, Nguyen QD, Robins E, Arstad E, Aboagye EO (2009). [18F]fluoromethyl-[1,2-2H4]-choline: a novel radiotracer for imaging choline metabolism in tumors by positron emission tomography. Cancer Res.

[15] Davies H, Bignell GR, Cox C, Stephens P, Edkins S, Clegg S, Teague J, Woffendin H, Garnett MJ, Bottomley W, Davis N, Dicks E, Ewing R, Floyd Y, Gray K, Hall S, Hawes R, Hughes J, Kosmidou V, Menzies A, Mould C, Parker A, Stevens C, Watt S, Hooper S, Wilson R, Jayatilake H, Gusterson BA, Cooper C, Shipley J, Hargrave D, Pritchard-Jones K, Maitland N, Chenevix-Trench G, Riggins GJ, Bigner DD, Palmieri G, Cossu A, Flanagan A, Nicholson A, Ho JW, Leung SY, Yuen ST, Weber BL, Seigler HF, Darrow TL, Paterson H, Marais R, Marshall CJ, Wooster R, Stratton MR, Futreal PA (2002). Mutations of the BRAF gene in human cancer. Nature.

[16] da Rocha Dias S, Friedlos F, Light Y, Springer C, Workman P, Marais R (2005). Activated B-RAF is an Hsp90 client protein that is targeted by the anticancer drug 17-allylamino-17-demethoxygeldanamicin. Cancer Res.

[17] Grbovic OM, Basso AD, Sawai A, Ye Q, Friedlander P, Solit D, Rosen N (2006). V600E B-Raf requires the Hsp90 chaperone for stability and is degraded in response to Hsp90 inhibitors. Proc Natl Acad Sci U S A.

[18] Chung YL, Troy H, Banerji U, Jackson LE, Walton MI, Stubbs M, Griffiths JR, Judson IR, Leach MO, Workman P, Ronen SM (2003). Magnetic resonance spectroscopic pharmacodynamic markers of the heat shock protein 90 inhibitor 17-allylamino,17-demethoxygeldanamycin (17AAG) in human colon cancer models. J Natl Cancer Inst.

[19] Solit DB, Garraway LA, Pratilas CA, Sawai A, Getz G, Basso A, Ye Q, Lobo JM, She Y, Osman I, Golub TR, Sebolt-Leopold J, Sellers WR, Rosen N (2006). BRAF mutation predicts sensitivity to MEK inhibition. Nature.

[20] Arias-Mendoza F, Payne GS, Zakian KL, Schwarz AJ, Stubbs M, Stoyanova R, Ballon D, Howe FA, Koutcher JA, Leach MO, Griffiths JR, Heerschap A, Glickson JD, Nelson SJ, Evelhoch JL, Charles HC, Brown TR (2006). In vivo 31P MR spectral patterns and reproducibility in cancer patients studied in a multi-institutional trial. NMR Biomed.

[21] Barba I, Cabanas ME, Arus C (1999). The relationship between nuclear magnetic resonance-visible lipids, lipid droplets, and cell proliferation in cultured C6 cells. Cancer Res.

[22] Podo F (1999). Tumour phospholipid metabolism. NMR Biomed.

